# A Possible Explanation for the Variable Frequencies of Cancer Stem Cells in Tumors

**DOI:** 10.1371/journal.pone.0069131

**Published:** 2013-08-07

**Authors:** Renato Vieira dos Santos, Linaena Méricy da Silva

**Affiliations:** 1 Departamento de Física, Instituto de Ciências Exatas, Universidade Federal de Minas Gerais, Belo Horizonte, Minas Gerais, Brasil; 2 Laboratório de Patologia Comparada, Instituto de Ciências Biológicas, Universidade Federal de Minas Gerais, Belo Horizonte, Minas Gerais, Brasil; Université de Nantes, France

## Abstract

A controversy surrounds the frequency of cancer stem cells (CSCs) in solid tumors. Initial studies indicated that these cells had a frequency ranging from 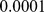 to 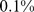 of the total cells. Recent studies have shown that this does not always seem to be the case. Some of these studies have indicated a frequency of 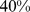. In this paper we propose a stochastic model that is able to capture this potential variability in the frequency of CSCs among the various type of tumors. Considerations regarding the heterogeneity of the tumor cells and its consequences are included. Possible effects on conventional treatments in clinical practice are also described. The model results suggest that traditional attempts to combat cancer cells with rapid cycling can be very stimulating for the cancer stem cell populations.

## Introduction

In recent years there has been increasing evidence for the *Cancer Stem Cell* (CSC) hypothesis [1]–[4], according to which tumor formation is a result of genetic and epigenetic changes in a subset of stem-like cells, also known as *tumor-forming* or *tumor-initiating* cells [5]. *Cancer stem cells* (CSCs) were first identified in leukemia and more recently in several solid tumors such as brain, breast, cervix and prostate tumors [4]. It has been suggested that these are the cells responsible for initiating and maintaining tumor growth [6]. In this paper, we study a model for tumor growth assuming the existence of cancer stem cells, or *tumor initiating cells* [6]–[8].

The conceptual starting point relevant to the CSC theory is constructed from the known tumor heterogeneity. We now know that cells in a tumor aren't all identical copies of each other, but that they display a striking array of characteristics [9]–[13]. The CSC theory recognizes this fact and develops its consequences. And one of the most immediate consequences for clinical practice is that conventional treatments can attack the wrong cell type. The appeal of the CSC idea can be described through the following analogy: just as killing the queen bee will lead to the demise of the hive, destroying cancer stem cells, should, in theory, stop the tumor from renewing itself. Unfortunately, things are never that simple. In the hive, workers react quickly to the death of queen by replacing her with a new one. And there is some evidence [8], [14] suggesting that the same may occur in a tumor due to a phenomenon known as *cell plasticity*, which allows differentiated tumor cells to turn into cancer stem cells, should the situation call for this. One goal of the present study is to evaluate the possible effects of this plasticity. Analogies with super organisms such as bee colonies are taken much more seriously in [15].

Stem cells in general (the same applies to CSCs) tend to be found on specific areas of a tissue where one particular microenvironment, called *niche* [16], [17], promotes the maintenance of their vital functions. Such a niche is specialized in providing factors that prevent differentiation and thus maintain the stemness of CSCs and, ultimately, the tumor's survival. Stem cells and niche cells interact with each other through adhesion molecules and paracrine factors. This complex network of interactions exchanges molecular signals and maintains the unique characteristics of stem cells, namely, pluripotency and self-renewal.

In this paper, we are interested in investigating a controversy related to the frequency in which CSCs appear in various tumors [18]–[25]. In the initial version of the CSC theory, it was believed that these cells were a tiny fraction of the total, ranging from 0.0001 to 0.1 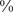 [26]. However, more recent studies have shown a strong dependence of the number of CSCs present in the tumor with the experimental xenograft model used. In explicit contrast to what was previously thought, in [27] a proportion of CSCs of approximately 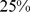 was observed. Other studies have confirmed this observation [26], [28], [29] with the possibility of a proportion of up to 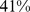 [30]. In [31] the authors provide evidence that this discrepancy may be due to the possibility of phenotypic switching between different tumor cells. Phenotypic switching is interpreted as the possibility of a more differentiated cancer cell being able to, under the appropriate conditions, dedifferentiate into cancer stem cell. This is the cellular plasticity mentioned above.

In [32] it is suggested that inconsistencies in the numbers of cancer stem cells reported in the literature can also be explained as a consequence of the different definitions used by different researchers. Different assays will give different numbers of cells, which can be orders of magnitude away from each other. Articles [31] and [32] provide different explanations for the discrepancy in the frequency of CSCs. Our arguments are consistent with the results of [31].

Considering that the complexity of the cellular microenvironment can be modeled by the insertion of a Gaussian noise into the equation that describes the population dynamics, we show that a noise-induced transition occurs. That corresponds to the emergence of a bimodal stationary probability distribution. This happens when the noise intensity 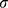 exceeds a critical limit value 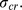


In this paper we show that *cell plasticity* [14], [33], [34], combined with a complex network of interactions modeled as noise, can induce discrepant (too small or too large) stationary CSC populations. Effects related to tumor heterogeneity and clinical treatments will be discussed at the end, occasion in which the model parameters possess the appropriate biological interpretations.

## Methods

### Model Assumptions

In the model used in this paper, cancer stem cells can perform three types of divisions, according to [35]:

- **symmetric self-renewal:** cell division in which both daughter cells have the characteristics of the mother stem cell, resulting in an expanding population of stem cells;
- **symmetric differentiation:** a stem cell divides into two progenitor cells;
- **asymmetric self-renewal** a cancer stem cell (denoted by *C*) is generated and a progenitor cell (mature cancer cell, denoted by *P*) is also produced;

We have developed a simple mathematical model for the stochastic dynamics of CSCs in which the three division types possess intrinsic replication rates, which are assumed to be time-independent. We assume, therefore, that besides the three described types of division, there is also the possibility of a transformation in which a progenitor cell can acquire characteristics of stem cells where, for all practical purposes, we may regard it as having become a dedifferentiated CSC. This hypothesis has experimental support [36]. These dedifferentiated cells do not become cancer stem cells, but rather develop CSC like behavior by re-activating a subset of genes highly expressed in normal hematopoietic stem cells [14]. The biological mechanisms underlying this transformation are described in [31], for example. As mentioned previously, we refer to this process as *cell plasticity*. Finally, we assume that cells are well mixed, so that we can ignore spatial effects.

The model proposed is a natural extension of what is proposed in [37]. We also incorporates the possibility of competition between CSCs and between the progenitor cells in order to limit the exponential growth of the linear model in [37]. This is described in the next subsection.

### The basic model

We assume that the dynamics of cancer stem cells (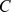) and progenitor cells (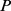) are governed by the following reactions:
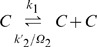


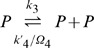


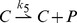


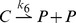


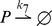


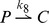
(1)


The first and second reactions, in the forward sense, models cell proliferation, which occurs at a rate of 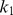 and 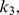 respectively. The constants 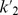 and 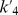 are associated with the reverse process and describe the intensity of competition between the CSCs and progenitors cells, respectively, and prevents their unlimited exponential growth. Many studies, experimental and theoretical, justify this approach [38]–[47]. As long as no mechanical nor nutritional restrictions apply, the tumor cells go on replicating with a constant duplication time. After a while, however, several constraints force the development of a necrotic core, and growth slows down towards some asymptotic level of saturation. 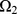 and 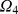 are constants related to the carrying capacity of the model. The third reaction involving 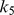 originates from the asymmetric transformation of CSCs in CSC daughter and progenitor cell types. The reaction involving the 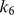 rate is related to a symmetrical division of the stem cell, which gives rise to two progenitor cells. The penultimate reaction is associated with the progenitor cell's death at rate 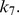 Finally, 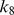 is the rate of dedifferentiation. All rates have dimension 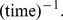 The specific time unit (months, quarters, years, etc.) will depend on the type and aggressiveness of the tumor.

Using the law of mass action, we can write
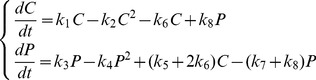
(2)with 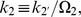 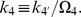 Setting 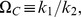 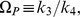 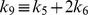 and 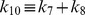 and making the substitutions 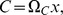 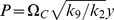 and 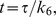 equation (2) can be written as (see Appendix S1)
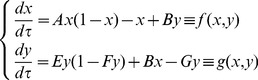
(3)with
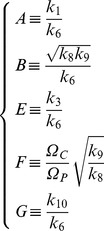
(4)As 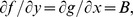 equation (3) represents a gradient system [48] with potential 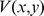 given by (see Appendix S1)



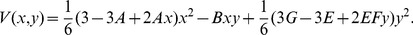
(5)As a consequence [49]:

1. The eigenvalues of the linearization of equation (3) evaluated at equilibrium point are real.
2. If 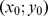 is an isolated minimum of 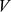 then 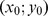 is an asymptotically stable solution of (3).
3. If 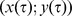 is a solution of (3) that is not an equilibrium point then 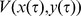 is a strictly decreasing function and is perpendicular to the level curves of 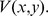

4. There are no periodic solutions of (3).

Sufficiently small 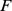 (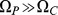) implies large differences in 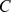 and 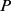 equilibrium populations. For parameters 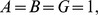 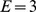 and 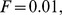 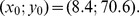 If we set 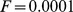 keeping the other parameters fixed, we have 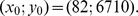


### Adiabatic elimination

The proposed model in (1) is in fact a general model of stem cells and does not carry any specific characteristic of cancer stem cells. All properties considered, such as plasticity and changes in the microenvironment conditions (to be included later), are also found in normal, stem cell tissue systems. The features associated with cancer stem cells are related to the large carrying capacity of progenitor cells when compared with the carrying capacity of CSCs. This fact is represented numerically by the choice of model parameters made below and is important because it allows a simplification using the adiabatic approximation.

**Figure 1:**
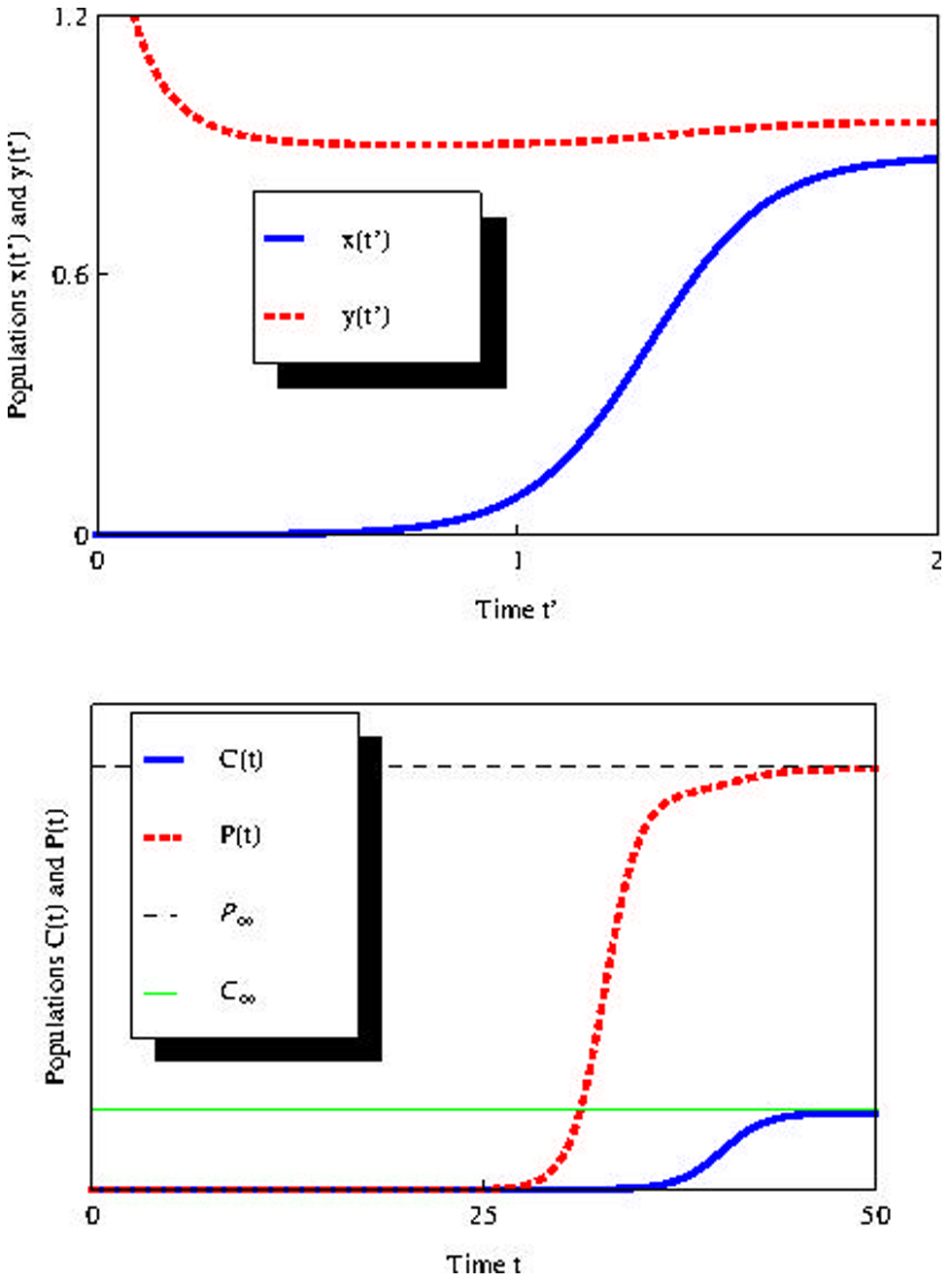
Numerical solutions of differential equations. **Top:** Numerical solution for reescaled equation (6). Horizontal axis is time 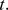 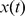 and 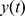 represent the rescaled population of cancer stem cells and progenitor cells, respectively. **Bottom:** Numerical solution for equation (2). 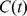 and 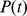 represent he population of cancer stem cells and progenitor cells, respectively. 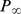 and 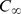 represent the limits of 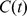 and 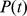 when 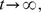 respectively. Parameters values: 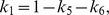 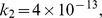 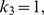 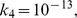 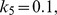 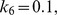 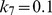 and 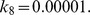 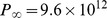 and 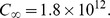 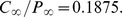

We can write (2) as (see Appendix S1)
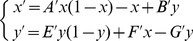
(6)with 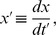 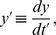 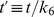 and



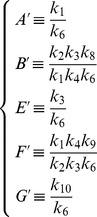
(7)
Figure (1) shows the numerical solutions of equations (6, Top) (the rescaled equation) and (2, Bottom) for the parameter values shown in table 1 (which correspond to 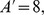 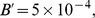 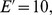 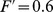 and 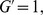 and 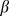 is a general parameter with dimension 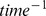 required for dimensional consistency in the following analysis):

**Table 1:** Parameter Values.

Parameters	*k*_1_	*k*_2_	*k*_3_	*k*_4_	*k*_5_	*k*_6_	*k*_7_	*k*_8_	β
Values	β-*k*_5_-*k*_6_	4×10^−13^	1	10^−13^	0.1	0.1	0.1	10^−5^	1

Considering the global rate 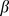 (we use 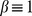 throughout the text) and assuming 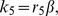 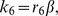 we make the usual assumption 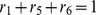 [50] and write 
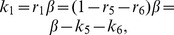
 where 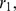 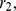 and 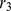 are probabilities. The values for 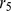 and 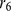 are consistent with those estimated in [50]. For these parameter values, 
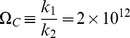
 and 
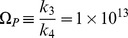
 (see Appendix S1). These are rescaled parameters in 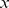 and 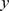 variables, respectively. Stationary values for 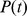 and 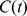 are 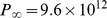 cells and 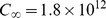 cells, respectively. Adjusting the 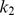 and 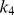 parameters, we can easily obtain more suitable values for the CSC and progenitor cell equilibrium populations, according to possible new experimental results.

Employing standard adiabatic elimination methods, we can write equation (6) as
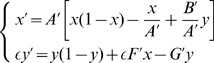
(8)where 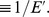 If we consider 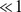 (this is equivalent to considering the progenitor cell division rate sufficiently large) we can perform adiabatic approximation [51], [52] in (8) and, setting 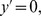 we obtain the following equation for 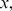 expanding in Taylor series up to first order in 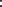


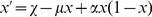
(9)where 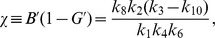 
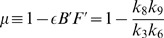
 and 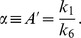 Note that 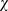 can be positive or negative depending on the magnitude of 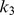 and 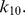


If we set a small enough value for  with respect to 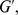 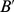 and 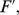 we can further simplify and write 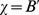 and 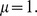 We observe that the plasticity phenomenon (associated with 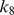) is crucial for the existence of the constant term 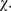 For this reason, from now on we will consider the parameter 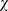 as representing the plasticity phenomenon in the reduced equation (9).

### The deterministic equation

For comparison with the stochastic study of the next section, we will briefly review the deterministic analysis of the problem. An analytic solution of Eq. (9) is possible. For the initial condition 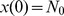, one has
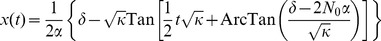
(10)with 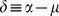 and 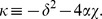 The physically relevant stable fixed point is



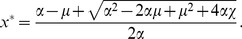
(11)The 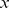 scaled population size dynamics can be thought of as analogous to the motion of a particle in a potential 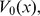 seeking its minimum point, with 
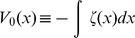
 with 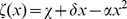 from (9). Thus, 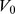 is given by the cubic polinomial,
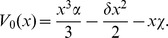



We see from (11) that by increasing either 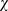 or 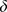, the minimum 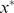 of 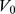 moves to the right in the potential, thus favoring CSCs population. Such behavior, of course, is expected, since an increase of 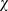 means an increase in frequency in which the induced plasticity mechanism occurs, and an increase of 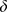 is an increase of the symmetric renewal rate of cancer stem cells, both of which increase the population.

## Results

### Noise in the CSCs niche

#### Environmental noise

In tumor tissue, the growth rate and other parameters are influenced by many environmental factors, *e.g.*, degree of vascularization of tissues, supply of oxygen and nutrients, immunological state of the host, chemical agents, gene expression, protein synthesis, mechanical stress, temperature, radiation, etc [50], [53]–[55]. Given the many perturbations affecting the CSC niche, we expect parameters such as growth rate to be random, rather than fixed, to give a more reliable description. We propose a simplification in the interaction mechanisms between cancer stem cells and their niche by adding an external Gaussian white noise in an attempt to capture the essential aspects of this complexity in a mathematically tractable way.

It is worth noting that in conjunction with nonlinear interactions, noise can induce many interesting phenomena, such as stochastic resonance [56], noise-induced phase transitions [57], noise-induced pattern formation, and noise-induced transport [51], [58].

#### Including external noise

To model the effect of external noise, focusing initially on the CSCs proliferation rate (by making 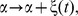 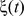 is the noise with the statistical properties described below), we modify the deterministic equation (9) as follows:
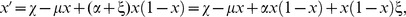
(12)where 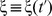 is a Gaussian white noise with statistical properties 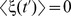 and 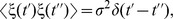 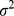 is the variance of 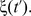 Furthermore, 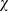 is considered a constant related to the plasticity phenomenon and 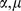 have interpretations similar to those of equation (9), where 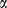 now represents the average symmetric division rate. The noise term in equation (12) represents fluctuations in parameter 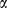, due to the complexity of the microenvironment, as discussed above. We include noise in this term because it is more important in the CSCs population dynamics, since it is this parameter that regulates symmetric reproduction 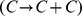. Later on we will add yet another noise in the plasticity constant.

We can write the Langevin equation (12) as a stochastic differential equation (considerations concerning the interpretation of the multiplicative term, *i.e.*, if Itô or Stratonovich or other, will be made below) in the form of
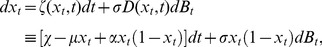
(13)where we define the drift 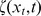 and diffusion 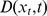 functions and where 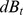 is the Wiener process increment [52], [59], [60]. The stationary probability distribution 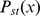 of the stochastic process defined by (13) is given by [52]

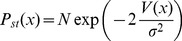
(14)where 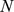 is a normalization constant and 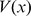 is the stochastic effective potential defined by
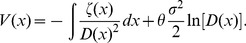
(15)Here 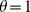 refers to the Stratonovich interpretation of (13) and 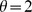 to the Itô version. Substituting the drift and diffusion functions, we get
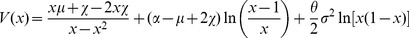
(16)and



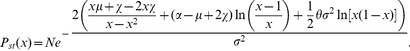
(17)The maximum 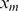 of 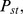 which corresponds to the minimum of 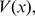 can be obtained from the following equation [61]:
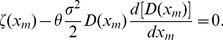
(18)


We see that for 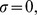 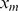 corresponds to the value given by 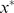 in eq. (11). From the drift and diffusion functions, we get:
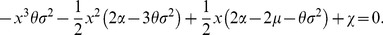
(19)


The condition for (19) possessing three real roots (corresponding to the two extremes of 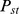) is [62]:
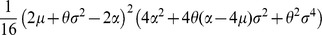


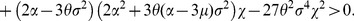
(20)


For example, for the parameters values 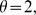 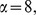 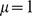 and 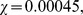 the critical value 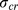 above which a transition is induced in 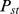 is 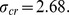


**Figure 2:**
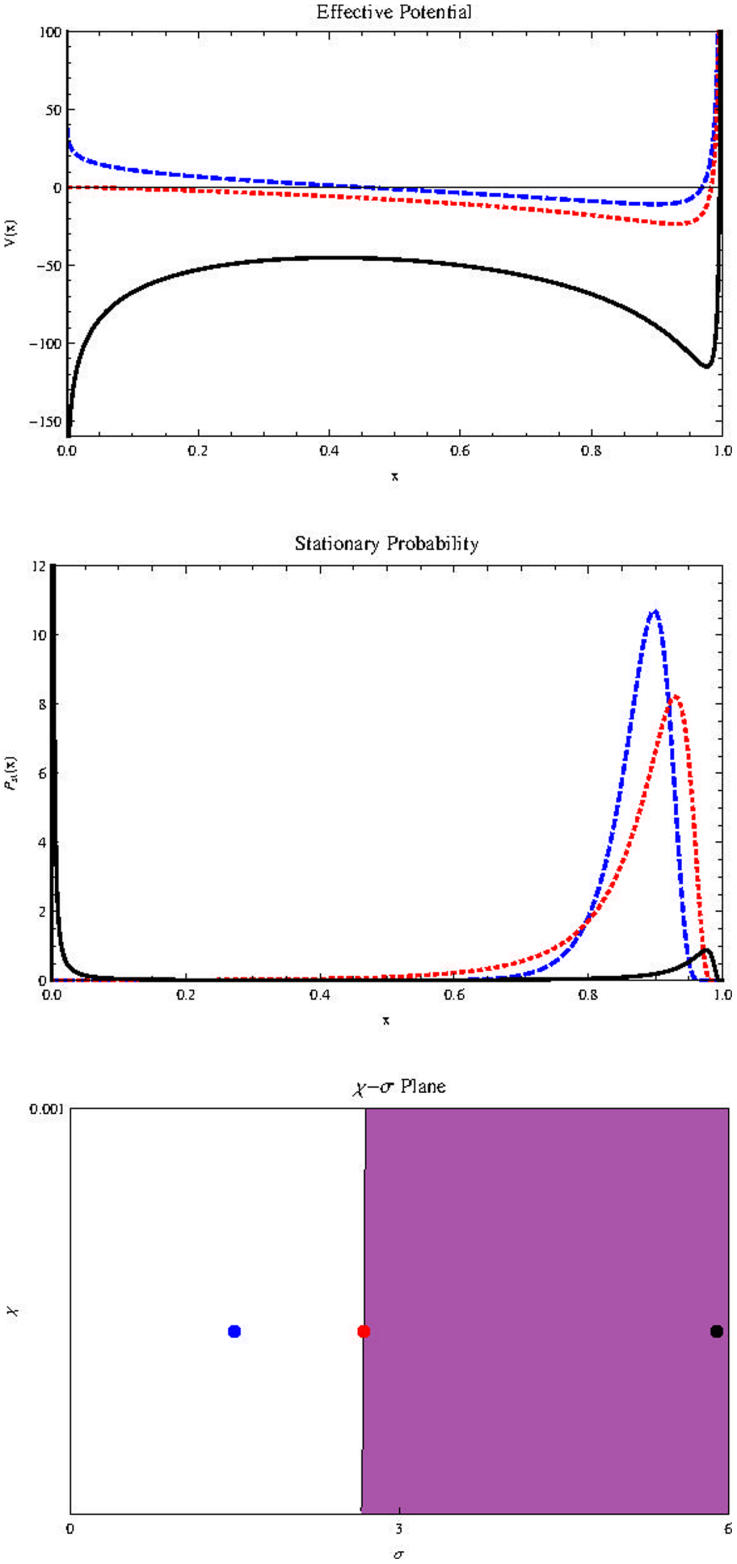
Effects of noise intensity on 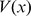 and 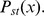. Effect of 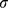 on 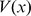 (on the top) and 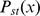 (in the middle) for parameters 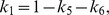 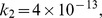 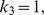 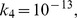 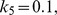 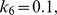 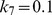 and 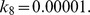 Horizontal axis represents population size 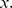 Blue, dashed curve: 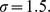 Red, dotted: 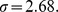 Black, thick: 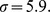 Below we also show the 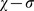 plane with 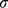 in the horizontal axis.

Figures (2) show, in Stratonovich interpretation (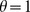), (the results do not change qualitatively if we use Itô. For a discussion quite enlightening about the controversial dilemma Itô/Stratonovich, see [63]) the effect of increasing the noise intensity in the stochastic effective potential 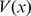 (Top) and in the stationary probability distribution 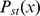 (Middle). Below is the 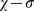 plane. The shaded region corresponds to high values of 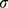 where 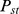 is bimodal. Note that the presence of plasticity (represented by 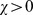) implies the survival of cells populations regardless of noise intensity. Inclusion of external noise can induces the appearance of a bimodal stationary probability distribution, which leads to a result quite different from the deterministic case: while the population in the deterministic case will necessarily reach the value 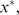 in the stochastic case the population is unlikely to reach 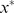 if 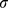 is above its critical value 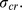 It is much more likely to possess a nonzero (if 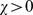), very small population (left peak of 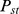) or a very large one (right peak of 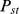). This peak positioned to the right is associated with a population near the maximum value 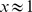 in the rescaled variable 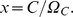 It stands for the possibility that the population of cancer stem cells possess a value close to 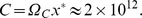 This represents a significant fraction of the population of progenitor cells 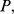 a fraction that depends mainly on the equilibrium value 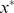 of the deterministic equation given by (11), never exceeding this threshold. When we insert noise in the plasticity 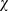 this is no longer the case.

**Figure 3:**
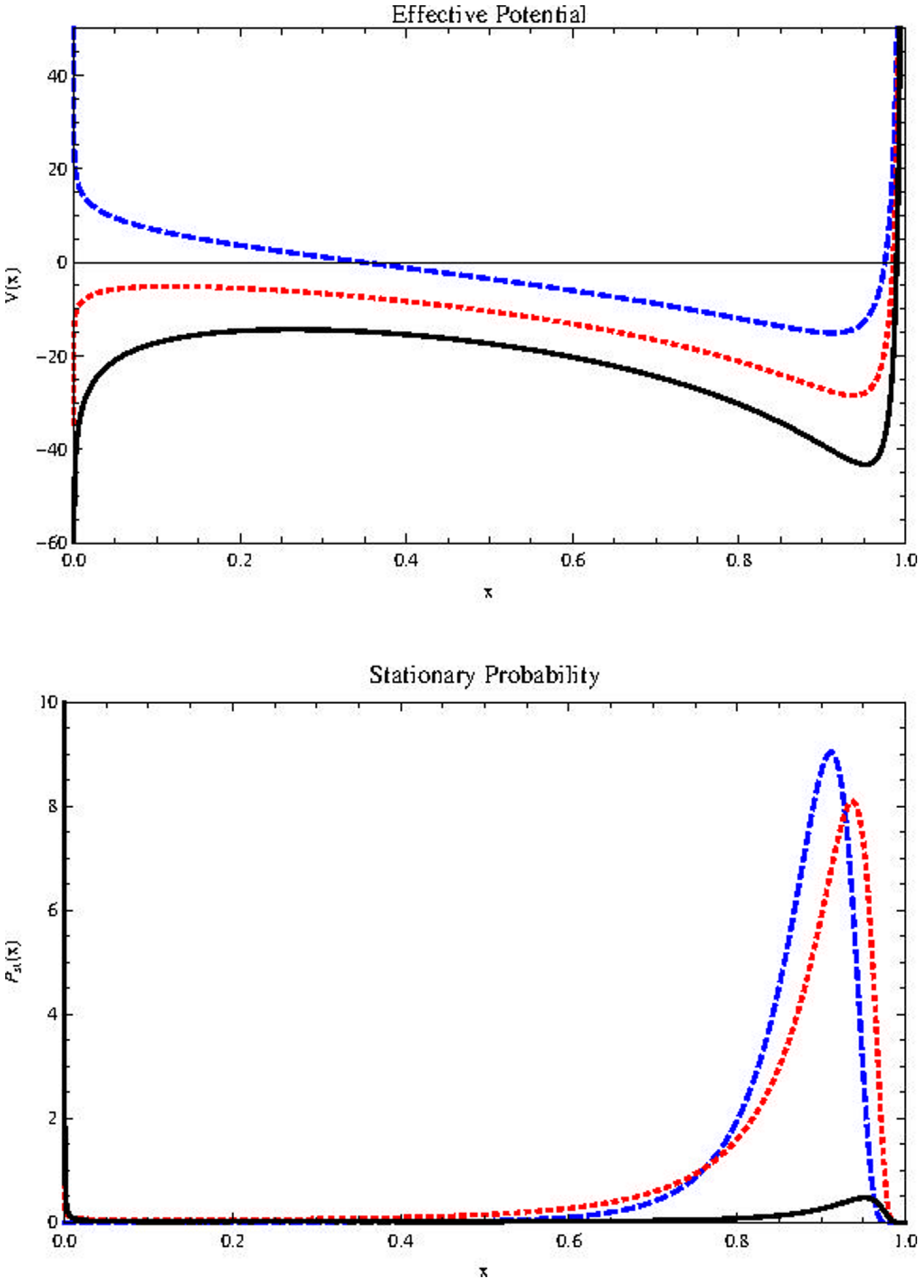
Effects of noise intensity on 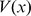 and 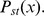. Effect of 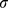 on 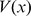 (on the top) and 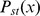 (at bottom) for 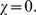 Horizontal axis represents population size 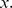 Blue, dashed curve: 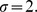 Red, dotted: 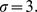 Black, thick: 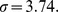 Other parameters are as in figure (2). For sufficiently high values of 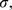 the CSCs population is extinguished.

The inhibition of the host's immune system, which can result in a decrease of the microenvironmental complexity, is equivalent in our model to a decrease of 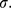 Therefore, a xenograft performed in immunosuppressed mice may, over time, present significantly large CSC populations. This may have been the case for the experiments conducted in [27]. On the other hand, the left peak in 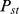 may represent a tiny fraction of the CSCs population, as commonly reported in the pioneering experiments mentioned in the introduction, in which less immunosuppressed mice were used. If 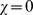 and 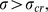 it is much more likely that the population becomes extinct as shown in figure (3).

**Figure 4:**
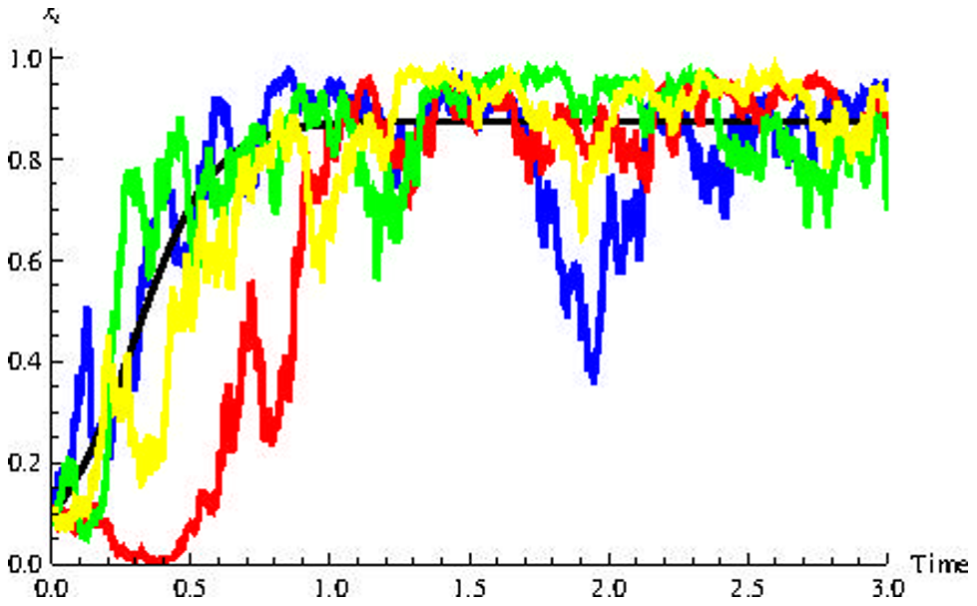
Some possible trajectories for the population dynamics with weak noise. The rugged curves show four realizations of stochastic process (13) with 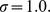 The black curve shows the deterministic case, 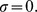

**Figure 5:**
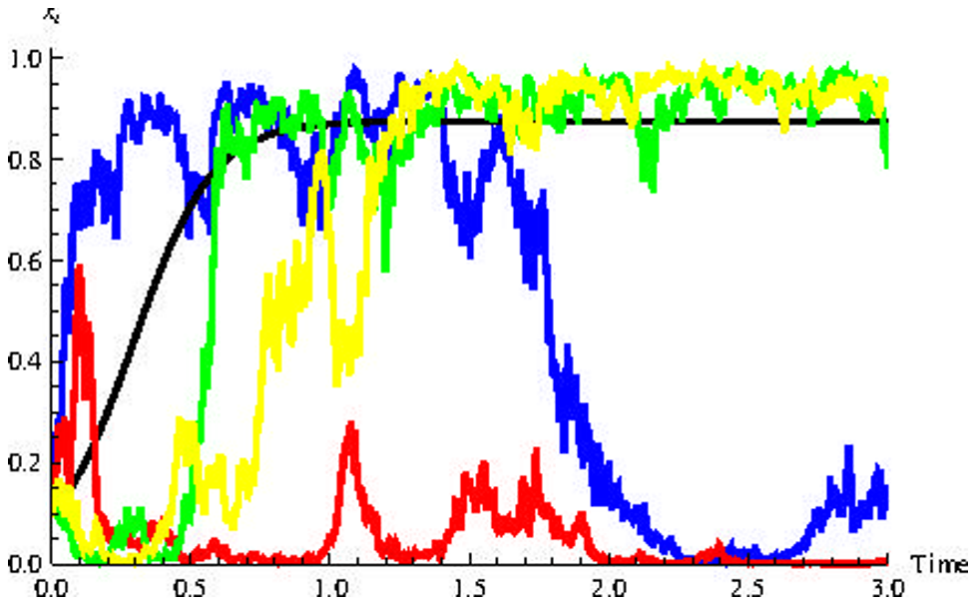
Some possible trajectories for the population dynamics with strong noise. The rugged curves show four realizations of stochastic process (13) with 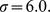 The black curve shows the deterministic case, 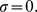 Some cases demonstrate the possibility of spontaneous remission.

Figures (4) and (5) show five trajectories of the relevant stochastic process, constructed using the Euler algorithm [64], with initial condition 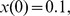 for 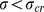 and 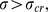 respectively. The black curve represents the solution for 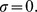 We see in Figure (5) that for high values of 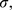 some trajectories can exhibit spontaneous regression of the CSCs. This seems plausible in light of the supporting evidence from many clinical reports [65].

**Figure 6:**
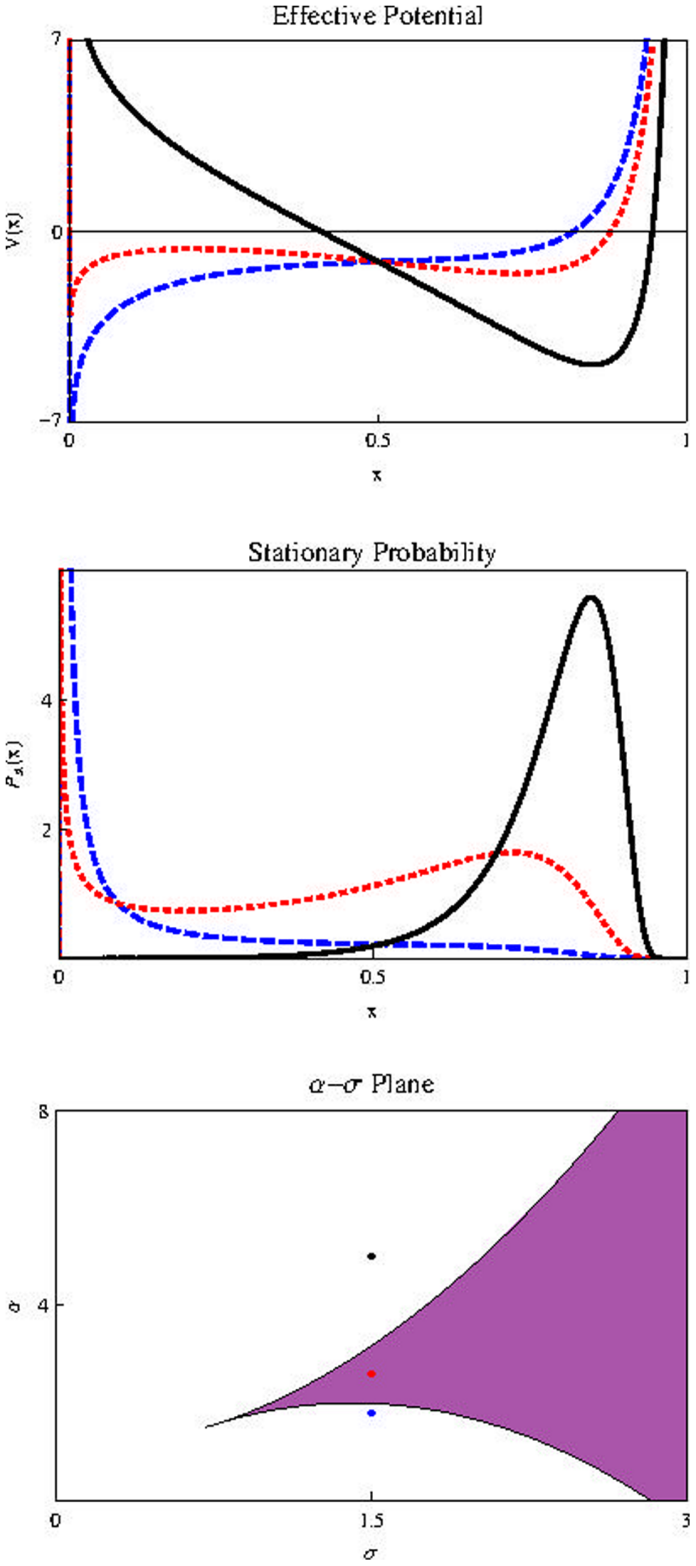
Effect of 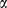 on 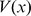 (on the top) and 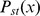 (in the middle) and the 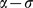 plane at bottom (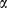 on the vertical axis and 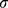 on the horizontal axis). The parameters are: 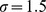 in all figures. Blue-dashed: 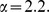 Red-dotted: 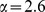 and Black-thick: 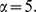

Figure (6) shows the effect of 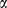 on 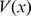 (Top) and 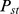 (Middle). Sufficiently small values of 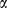 refer to unimodal distributions with left asymmetry (blue curve/dot). Intermediate values correspond to bimodal distributions (shaded area in the 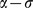 plane, red curve/dot). Sufficiently high levels of 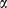 correspond to unimodal distributions with right asymmetry (black curve/dot).

We conclude in this section that the cell plasticity phenomenon is necessary for the existence of a cancer stem cell population as a small fraction of total tumor cells. Of course, microenvironmental conditions consistent with high noise levels are also necessary.

### Colorful background noise

We can ask ourselves what effects the variability induced by noise in 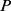 cells produce in the 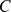 population. In equation (9), reminiscences of the presence of 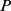 cells are manifested by the presence of 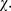 We can imagine this term as representing a source of background noisy for 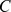 cells. The question that immediately arises is: what are the effects of a noise on the proliferation rate 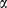 combined with other noise related to the plasticity in constant 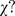 To answer this question, let's add the noise 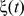 and 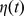 as 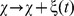 and 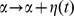 and write the equations
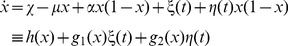
(21)

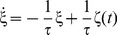
(22)where 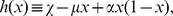 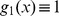 and 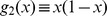 and 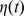 and 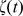 are white noises with the following properties



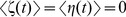
(23)

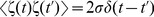
(24)

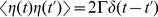
(25)

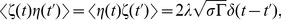
(26)where 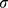 and 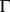 are the noise intensity of 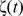 and 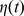 respectively, and 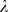 is the correlation between noises. Equation (22) represents the Ornstein-Uhlenbeck process that displays exponential correlation function described in equation (27) below with correlation time 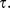 This stochastic process is called “colored noise”.

The two dimensional Markovian process defined by equations (21)–(26) is stochastically equivalent to the one-dimensional non-Markovian process described by (21), (24) and (25), with Gaussian colored noise 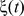 [52]:
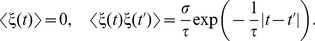
(27)


We are considering the possibility of a colored noise in 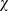 (for correlation time 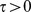). Thus we intend to capture the effects of noise in the plasticity more realistically.

Following [66], the stationary probability distribution is given by
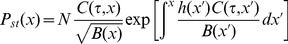
(28)where 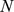 is a normalization constant and 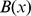 and 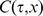 are given by



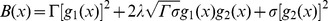



and
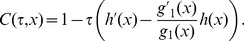



**Figure 7:**
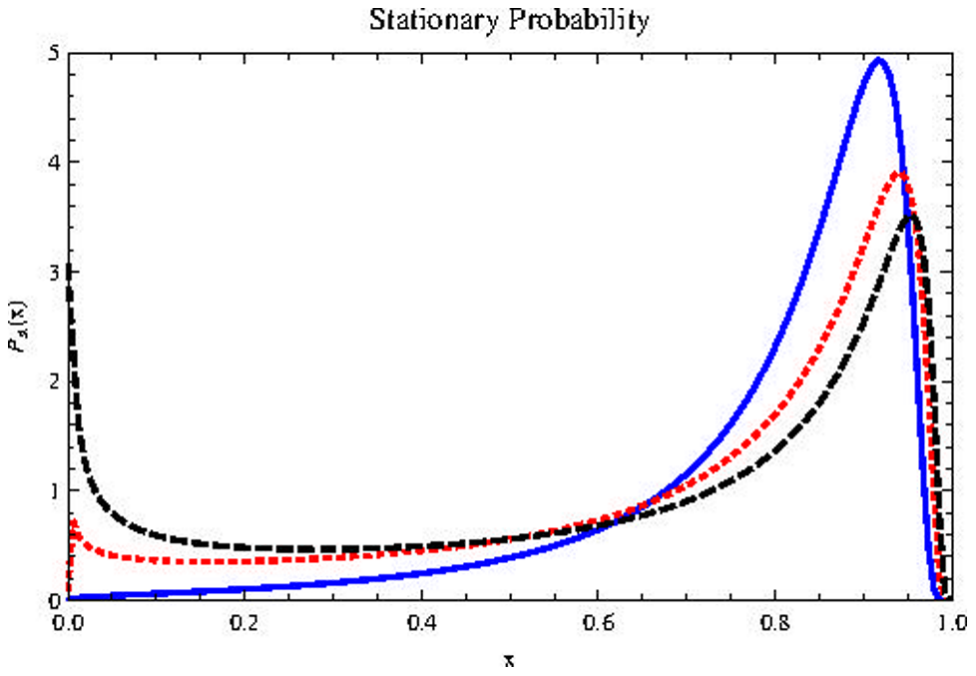
Stationary probability distribution for different values of 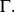. 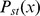
 with parameters 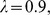 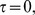 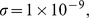 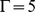 (blue), 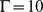 (red dotted) and 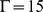 (black, dashed). Horizontal axis represents population size 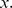 Fluctuations in the progenitor population 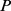 can stimulate CSCs extinction.

**Figure 8:**
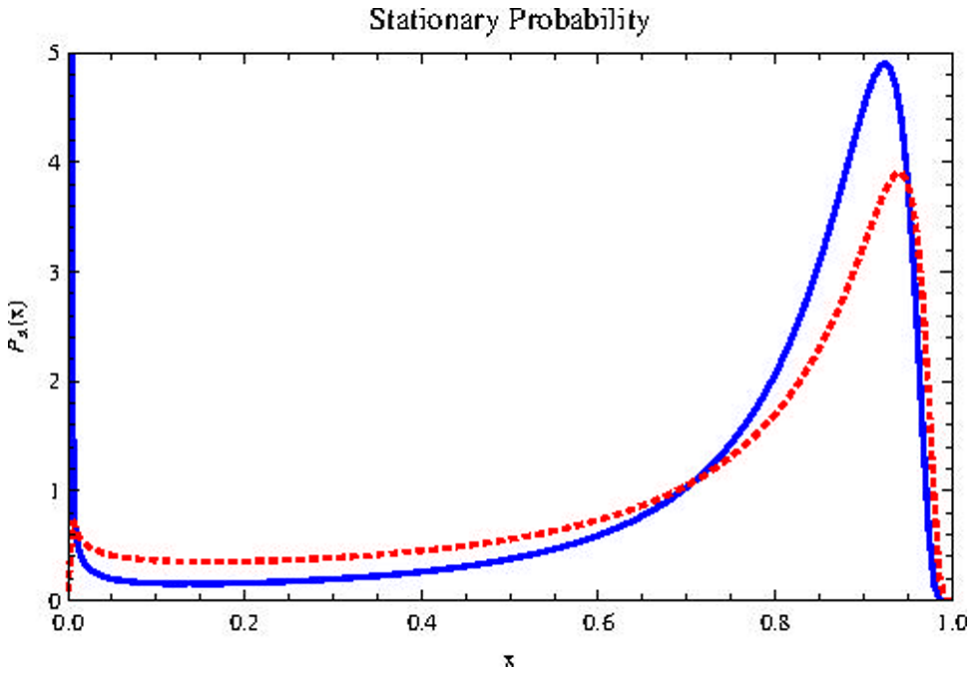
Dependence of 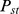 with 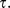. 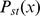
 with parameters 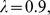 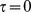 (red curve), 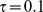 (blue curve), 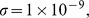 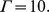 Horizontal axis represents population size 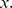 High values of 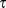 facilitates CSCs extinction.

In figure (7) we show the stationary probability distribution with 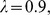 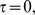 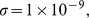 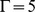 (blue), 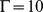 (red, dotted) and 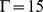 (black, dashed). Now we see that even for very small 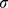 (the background noise intensity due to 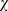), extinction of CSCs is possible for sufficiently high 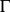 (the noise due to 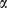), which does not occur when 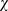 is deterministic. For 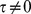 this statement becomes more evident, as shown in figure (8) where we used the same parameter values of previous figure with 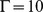 except that 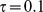 for blue thick curve and 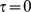 for red dotted curve. The conclusion is that the induction of fluctuations in the population of progenitor cells (represented by the background noise due to 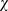) can promote CSC extinction.

### Some remarks on the interpretation of 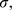 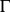 and 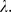


Before we continue the discussion about the effects of background noise, we will make some considerations about the interpretation that we assign to the parameters 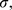 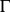 and 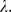


#### About 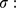


Given equation (9), we can interpret the system formed by CSCs as an isolated system that exchanges “particles” (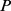 cells) with the external environment and “feels” the disturbances of the medium through the parameter 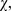 the window of communication with the outside. The intensity of these external disturbances is represented by parameter 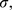 and 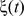 can therefore be interpreted as an external noise, external to the system formed by CSCs. When the body of the tumor is subjected to the effects of clinical treatments such as radiotherapy, chemotherapy or thermotherapy [67], the increase in the intensity of this parameter can be considerable.

#### About 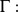


The direct contact of CSCs with their immediate microenvironment (their niche) is what enables exchange of nutrients and complex biochemical interactions that allow for cell life. Variability in this context represented by 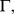 can be interpreted as an internal noise (internal noise here is not related in any way to the internal demographic noise as modeled by master equations). This internal noise affects the cell proliferation rate 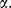


#### About 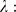


A very important aspect about cancer, as mentioned in the introduction, is that tumors contain heterogeneous populations of cells, which may contribute differently in extent and mechanism to the progression of malignancy [68]. Tumor heterogeneity is possibly one of the most significant factors that most treatment methods fail to address sufficiently. While a particular drug may exhibit initial success, the eventual relapse into tumor growth is due in many cases to subpopulations of cancer cells that are either not affected by the drug mechanism, possess or acquire a greater drug resistance, or have a localized condition in their microenvironment that enables them to evade or withstand the treatment. These various subpopulations may include cancer stem cells, mutated clonal variants, and tumor-associated stromal cells, in addition to cells experiencing a spatially different condition such as hypoxia within a diffusion-limited tumor region.

This important aspect is related to different forms in which the various sub-populations respond to various types of internal and external stimuli. Thus, we argue that the correlation coefficient 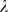 between the noise acts as a measure of this heterogeneity between the two populations we are considering. Since each noise is related primarily to a specific cell type, we have that parameter 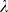 “measured” different responses of these cells to these stimuli. If the different subpopulations behave more or less in the same manner when subjected to various stimuli (low heterogeneity), 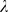 tends to approach 1. If the behaviors are independent, 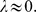 If the responses to the stimuli tend to be opposite (great heterogeneity), 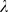 tends to approach −1.

**Figure 9:**
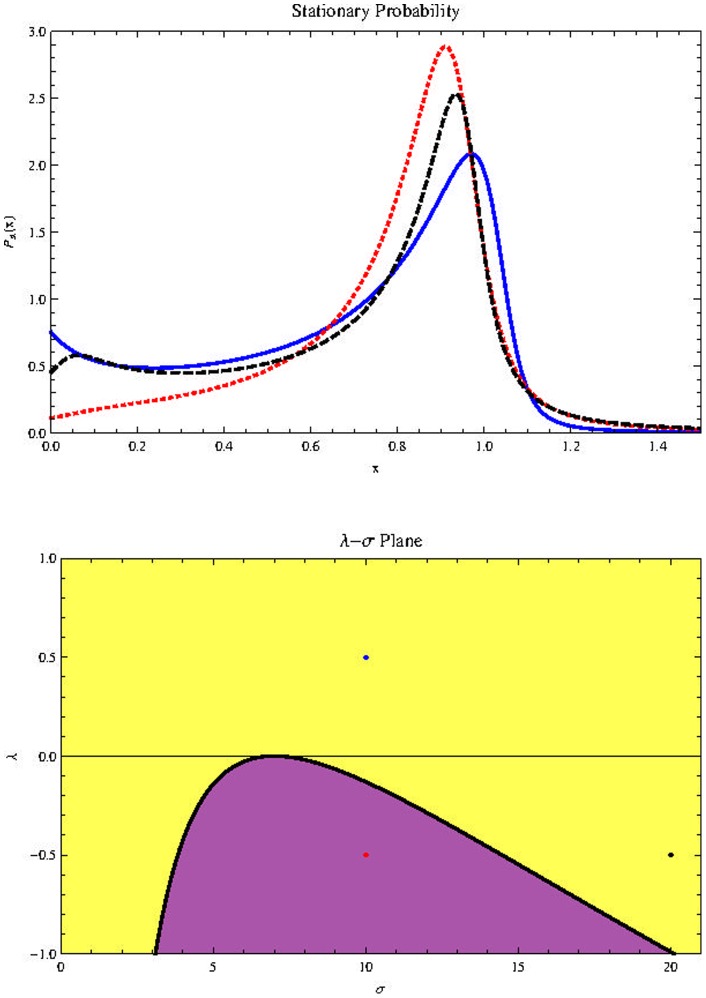
Effect of 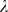 on 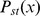 (top) with parameters 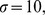 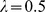 (Blue, thick line), 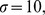 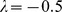 (Red, dotted line), 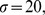 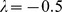 (Black, dashed line), 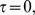 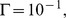 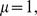 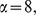 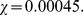. Horizontal axis represents population size 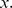 Bottom: 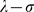 plane with 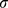 in the horizontal axis.

Figure (9) (Top) shows the possible effect of changes in 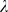 in stationary probability distribution for the parameters values shown in the description. The results for 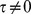 are analogous. Below is the 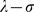 diagram. In the yellow region the stationary probability distribution is bimodal. We see that negative values of 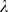 favor the survival of cancer stem cells. This result is no surprise, since it is known that the heterogeneity of the tumor provides the phenotypic variation required for natural selection to act to increase the robustness (a property that allows a system to mantain its function despite internal and external perturbations) of the tumor [10].

### Possible effect of conventional treatments

The proposed model in this paper is idealized and highly simplified. In addition, it does not rely on biological data for some values of the 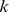 parameters. Therefore, the conclusions we can get from it in this section are merely theoretical speculations. Having said this, let's try to estimate the effects that conventional treatments may have on the CSC population.

In the proposed model we imagine that such treatments work directly on progenitor cells, since such treatments are designed to act mainly in cells that reproduce faster [69]. Thus, the effect on CSCs is indirect via background noise in a manner that is analogous to what was discussed above. Now we have the possibility of noise intensity 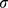 being much larger. Treatments act to eliminate progenitor cells and the tendency, therefore, is for parameter 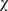 to approach zero. Since this is the parameter that connects the “underlying world” of cancer stem cells to the world of progenitor cells, we could imagine that the contact between the worlds is lost. This is no problem, however, because now we think of the background noise as an additive noise that arises as a result of external perturbations to the CSCs. Thus, we can consider equation (21) with 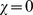 and think about the noise 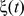 as is commonly understood when you introduce an additive noise in the equations “phenomenologically” or “by hand”.

**Figure 10:**
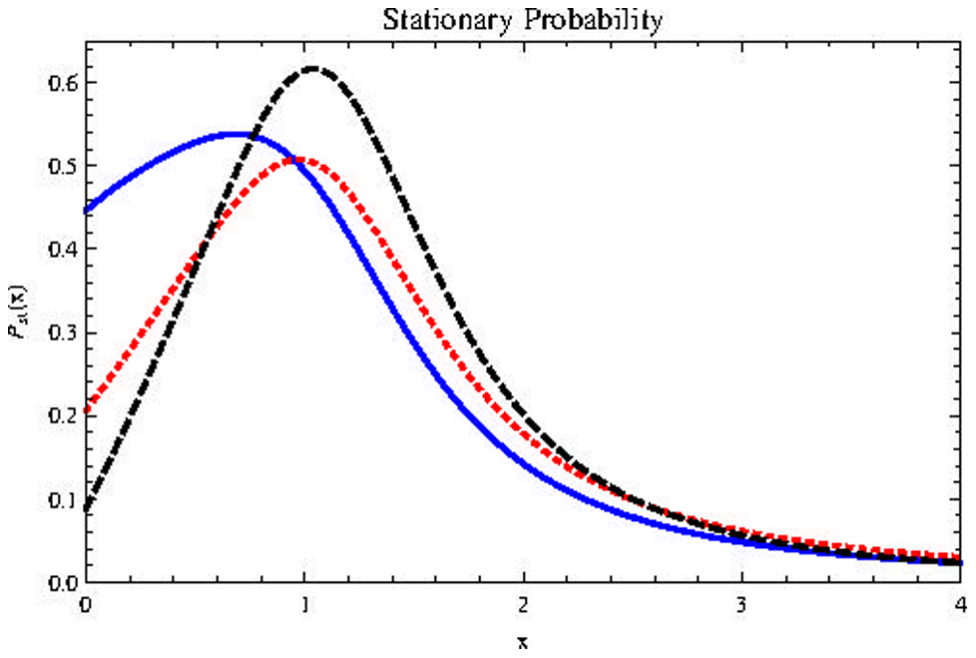
Effect of 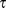 on 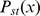 with parameters 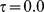 (Blue, thick line), 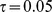 (Red, dotted line), 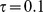 (Black, dashed line), 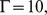 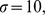 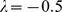 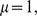 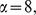 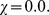 Horizontal axis represents population size 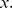

For large values of 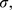 the parameter of greater relevance is 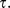 Figure (10) shows the effect on the stationary probability distribution: Positive values, even small ones, help cancer stem cells considerably not going extinct. The most important, however, is another fact, which is explicitly shown in this figure: The main consequence of exploring the possibility of an intense additive noise is that the population of cancer stem cells may be considerably greater than the maximum population of the deterministic model 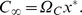 This means that the effects of conventional treatments that act primarily in the fast cycling cells, here represented by progenitor cells, can be extremely exciting for CSC proliferation. *Cancer stem cells enjoy noise*.

## Discussion

The importance of cellular plasticity in the conclusions we have drawn so far, is evident. In [32] the authors point out potential conceptual difficulties associated with the phenotypic switching hypothesis. They argue that if cancer cells can turn into cancer stem cells, then the very notion of CSC becomes blurred, since in this way the cancer cells could dedifferentiate at any time and acquire the potential immortality of CSCs. In the authors words, “the distinction between phenotypic switching and the original conventional model, run the risk of becoming purely semantic.” From a clinical perspective, this means that the existence or not of the CSCs is irrelevant, since we must try to kill all tumor cells and not just focus on tumor initiating cells. However, the fact that we have to kill the greatest possible amount of tumor cells does not mean that we have to try to do it in the same way for all of them. In [70], a near-twofold reduction in the density of brain tumors in mice was observed when authors combined standard anticancer drugs with the selective killing of CSCs, if compared with standard agents alone. With regard to the phenotypic switching property, selectively killing a population of CSCs can make room for progenitor cells to dedifferentiate and occupy this vacant niche space. Trying to limit “stemness” instead, by changing conditions of the niche that supports the life of CSCs, may be a more promising therapeutic strategy. This idea is in line with what is thought to be necessary for major mass extinctions [71].

Until now the properties of cancer stem cells were tested only in transplantation assays and their very existence have been questioned several times [6]–[8]. In [72], the authors use a lineage tracing technique that allows permanent, *in vivo* fluorescent marking of stem cells and their progeny, trying to put an end to the controversy of the existence of cancer stem cells in solid tumors. They unraveled the *in vivo* mode of tumor growth in its native environment and found that the majority of labeled tumor cells in benign skin tumors have only limited proliferative potential, whereas a fraction has the capacity to persist in the long term, giving rise to progeny that occupies a significant part of the tumor. Progression to cancer in benign skin tumors was associated with expansion of the CSC population and a decrease in the production of non-stem cells. This suggests that tumor evolution enriches the CSC population. Designing therapies that prevent increases in stemness may be a means to restrict tumor progression into cancer.

## Conclusion

We propose a model to describe the population dynamics of cancer cells, using the theory of cancer stem cells (CSCs). Our analysis allows us to address a controversy related to the frequency of such cells in tumors. Initially it was thought that these cells were relatively rare, comprising at most 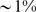 of the cancer cell population. More recent experiments, however, suggest that the CSC population need not be small. Taking into account the cellular plasticity property, which permits more mature cells to dedifferentiate into cells with characteristics of stem cells, we show that the discrepancy observed in the frequency of these cells is entirely consistent with the original hypothesis of the existence of cancer stem cells, as long as favorable conditions related to the complexity of the microenvironment are met. We assume that these conditions can be described by the inclusion of noise in the rate of tumor growth or in the rate at which the plasticity phenomenon occurs.

In the model where we take into account only the noise in the rate of CSC proliferation, we conclude that there is the possibility of the stationary probability distribution being bimodal. In the model that also incorporates noise in parameter 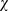 associated to the cellular plasticity phenomenon, the possibility of extinction arises and the fraction of CSCs in the tumor can assume quite high values, exceeding the threshold 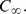 The “color” of this noise stimulates the CSC population. The correlation coefficient between noises is interpreted as a measure of heterogeneity between progenitor cells and cancer stem cells, since different cells respond to stimuli in different ways. This heterogeneity also excites the CSC population.

In future work we plan to extend the model to include spatial distribution. We will also investigate the possibility of a model based on a master equation to investigate the effects of demographic stochasticity.

## Supporting Information

Appendix S1 **Appendix to a possible explanation for the variable frequencies of cancer stem cells in tumors.**
(PDF)

## References

[1] Reya T, Morrison SJ, Clarke MF, Weissman IL (2001) Stem cells, cancer, and cancer stem cells. Nature 414: 105–111. 11689955 10.1038/35102167

[2] Clarke MF, Fuller M (2006) Stem cells and cancer: Two faces of eve. Cell 124: 1111–1115. 16564000 10.1016/j.cell.2006.03.011

[3] Vermeulen L, Sprick MR, Kemper K, Stassi G, Medema JP (2008) Cancer stem cells – old concepts, new insights. Cell Death and Differentiation aop. 10.1038/cdd.2008.20 18259194

[4] Dalerba P, Cho RW, Clarke MF (2007) Cancer stem cells: models and concepts. Annual review of medicine 58: 267–284. 10.1146/annurev.med.58.062105.204854 17002552

[5] Bomken S, Fišer K, Heidenreich O, Vormoor J (2010) Understanding the cancer stem cell. British journal of cancer 103: 439–445. 20664590 10.1038/sj.bjc.6605821 PMC2939794

[6] Lewis M (2008) Faith, heresy and the cancer stem cell hypothesis. Future oncology (London, England) 4: 585. 10.2217/14796694.4.5.585 PMC2577595 18922113

[7] Hill RP (2006) Identifying cancer stem cells in solid tumors: case not proven. Cancer Research 66: 1891–1895; discussion 1890. 10.1158/0008-5472.CAN-05-3450 16488984

[8] Welte Y, Adjaye J, Lehrach HR, Regenbrecht CR (2010) Cancer stem cells in solid tumors: elusive or illusive? Cell Commun Signal 8: 6. 20459772 10.1186/1478-811X-8-6 PMC2880310

[9] Denison TA, Bae YH (2012) Tumor heterogeneity and its implication for drug delivery. Journal of Controlled Release. 10.1016/j.jconrel.2012.04.014 PMC3421061 22537887

[10] Tian T, Olson S, Whitacre J, Harding A (2011) The origins of cancer robustness and evolvability. Integr Biol 3: 17–30. 10.1039/c0ib00046a 20944865

[11] Shackleton M, Quintana E, Fearon E, Morrison S (2009) Heterogeneity in cancer: cancer stem cells versus clonal evolution. Cell 138: 822–829. 19737509 10.1016/j.cell.2009.08.017

[12] Marusyk A, Polyak K (2010) Tumor heterogeneity: causes and consequences. Biochimica et Biophysica Acta (BBA)-Reviews on Cancer 1805: 105–117. 19931353 10.1016/j.bbcan.2009.11.002 PMC2814927

[13] Marusyk A, Almendro V, Polyak K (2012) Intra-tumour heterogeneity: a looking glass for cancer? Nature Reviews Cancer. 10.1038/nrc3261 22513401

[14] Rapp UR, Ceteci F, Schreck R (2008) Oncogene-induced plasticity and cancer stem cells. Cell Cycle 7: 45. 18196970 10.4161/cc.7.1.5203

[15] Grunewald T, Herbst S, Heinze J, Burdach S (2011) Understanding tumor heterogeneity as functional compartments-superorganisms revisited. Journal of translational medicine 9: 79. 21619636 10.1186/1479-5876-9-79 PMC3118334

[16] Lander A, Kimble J, Clevers H, Fuchs E, Montarras D, et al (2012) What does the concept of the stem cell niche really mean today? BMC biology 10: 19. 22405133 10.1186/1741-7007-10-19 PMC3298504

[17] Iwasaki H, Suda T (2009) Cancer stem cells and their niche. Cancer science 100: 1166–1172. 19432904 10.1111/j.1349-7006.2009.01177.x PMC11158862

[18] Ishizawa K, Rasheed Z, Karisch R, Wang Q, Kowalski J, et al (2010) Tumor-initiating cells are rare in many human tumors. Cell stem cell 7: 279–282. 20804964 10.1016/j.stem.2010.08.009 PMC2945729

[19] Stewart J, Shaw P, Gedye C, Bernardini M, Neel B, et al (2011) Phenotypic heterogeneity and instability of human ovarian tumor-initiating cells. Proceedings of the National Academy of Sciences 108: 6468. 10.1073/pnas.1005529108 PMC3081039 21451132

[20] Vargaftig J, Taussig D, Griessinger E, Anjos-Afonso F, Lister T, et al.. (2011) Frequency of leukemic initiating cells does not depend on the xenotransplantation model used. Leukemia. 10.1038/leu.2011.250 PMC3272414 21926966

[21] Sarry J, Murphy K, Perry R, Sanchez P, Secreto A, et al (2011) Human acute myelogenous leukemia stem cells are rare and heterogeneous when assayed in nod/scid/il2rãc-deficient mice. The Journal of Clinical Investigation 121: 384. 21157036 10.1172/JCI41495 PMC3007135

[22] Zhong Y, Guan K, Zhou C, Ma W, Wang D, et al (2010) Cancer stem cells sustaining the growth of mouse melanoma are not rare. Cancer letters 292: 17–23. 19944522 10.1016/j.canlet.2009.10.021

[23] Baker M (2008) Melanoma in mice casts doubt on scarcity of cancer stem cells. Nature 456: 553. 19052589 10.1038/456553a

[24] Johnston M, Maini P, Jonathan Chapman S, Edwards C, Bodmer W (2010) On the proportion of cancer stem cells in a tumour. Journal of theoretical biology 266: 708–711. 20678505 10.1016/j.jtbi.2010.07.031

[25] Baker M (2008) Cancer stem cells, becoming common. Nature Reports Stem Cells.

[26] Schatton T, Murphy G, Frank N, Yamaura K, Waaga-Gasser A, et al (2008) Identification of cells initiating human melanomas. Nature 451: 345–349. 18202660 10.1038/nature06489 PMC3660705

[27] Quintana E, Shackleton M, Sabel M, Fullen D, Johnson T, et al (2008) Efficient tumour formation by single human melanoma cells. Nature 456: 593–598. 19052619 10.1038/nature07567 PMC2597380

[28] Kelly P, Dakic A, Adams J, Nutt S, Strasser A (2007) Tumor growth need not be driven by rare cancer stem cells. Science 317: 337. 17641192 10.1126/science.1142596

[29] Williams R, Den Besten W, Sherr C (2007) Cytokine-dependent imatinib resistance in mouse bcr-abl+, arf-null lymphoblastic leukemia. Genes & development 21: 2283. 17761812 10.1101/gad.1588607 PMC1973142

[30] Boiko A, Razorenova O, van de Rijn M, Swetter S, Johnson D, et al (2010) Human melanomainitiating cells express neural crest nerve growth factor receptor cd271. Nature 466: 133–137. 20596026 10.1038/nature09161 PMC2898751

[31] Gupta P, Chaffer C, Weinberg R (2009) Cancer stem cells: mirage or reality? Nature medicine 15: 1010–1012. 10.1038/nm0909-1010 19734877

[32] Zapperi S, La Porta CAM (2012) Do cancer cells undergo phenotypic switching? the case for imperfect cancer stem cells markers. Scientific reports. 10.1038/srep00441 PMC3369193 22679555

[33] Chaffer C, Brueckmann I, Scheel C, Kaestli A, Wiggins P, et al (2011) Normal and neoplastic nonstem cells can spontaneously convert to a stem-like state. Proceedings of the National Academy of Sciences 108: 7950. 10.1073/pnas.1102454108 PMC3093533 21498687

[34] Strauss R, Hamerlik P, Lieber A, Bartek J (2012) Regulation of stem cell plasticity: Mechanisms and relevance to tissue biology and cancer. Molecular Therapy. 10.1038/mt.2012.2 PMC3345979 22314288

[35] Morrison S, Kimble J (2006) Asymmetric and symmetric stem-cell divisions in development and cancer. Nature 441: 1068–1074. 16810241 10.1038/nature04956

[36] Leder K, Holland E, Michor F (2010) The therapeutic implications of plasticity of the cancer stem cell phenotype. PloS one 5: e14366. 21179426 10.1371/journal.pone.0014366 PMC3003707

[37] Turner C, Stinchcombe AR, Kohandel M, Singh S, Sivaloganathan S (2009) Characterization of brain cancer stem cells: a mathematical approach. Cell Prolif 42: 529–40. 19555427 10.1111/j.1365-2184.2009.00619.x PMC6496718

[38] Laird AK (1964) Dynamics of tumour growth. British journal of cancer 18: 490. 10.1038/bjc.1965.32 PMC2071357 14316202

[39] Choe SC, Zhao G, Zhao Z, Rosenblatt JD, Cho HM, et al.. (2011) Model for in vivo progression of tumors based on co-evolving cell population and vasculature. Scientific reports 1. 10.1038/srep00031 PMC3216518 22355550

[40] Gliozzi AS, Guiot C, Delsanto PP (2009) A new computational tool for the phenomenological analysis of multipassage tumor growth curves. PloS one 4: e5358. 19396358 10.1371/journal.pone.0005358 PMC2670507

[41] Herman AB, Savage VM, West GB (2011) A quantitative theory of solid tumor growth, metabolic rate and vascularization. PloS one 6: e22973. 21980335 10.1371/journal.pone.0022973 PMC3182997

[42] Vaidya VG, Alexandro FJ Jr (1982) Evaluation of some mathematical models for tumor growth. International Journal of Bio-Medical Computing 13: 19–35. 7061168 10.1016/0020-7101(82)90048-4

[43] Weedon-Fekjær H, Lindqvist BH, Vatten LJ, Aalen OO, Tretli S, et al (2008) Breast cancer tumor growth estimated through mammography screening data. Breast Cancer Res 10: R41. 18466608 10.1186/bcr2092 PMC2481488

[44] Guiot C, Delsanto PP, Carpinteri A, Pugno N, Mansury Y, et al (2006) The dynamic evolution of the power exponent in a universal growth model of tumors. Journal of theoretical biology 240: 459–463. 16324717 10.1016/j.jtbi.2005.10.006

[45] Guiot C, Degiorgis PG, Delsanto PP, Gabriele P, Deisboeck TS (2003) Does tumor growth follow a universal law? Journal of theoretical biology 225: 147–151. 14575649 10.1016/s0022-5193(03)00221-2

[46] Castorina P, Zappalà D (2006) Tumor gompertzian growth by cellular energetic balance. Physica A: Statistical Mechanics and its Applications 365: 473–480.

[47] Von Bertalanffy L (1957) Quantitative laws in metabolism and growth. The quarterly review of biology 32: 217–231. 13485376 10.1086/401873

[48] Perko L (2000) Differential Equations and Dynamical Systems. Texts in Applied Mathematics. Springer.

[49] Hirsch M, Smale S, Devaney R (2004) Differential Equations, Dynamical Systems, and an Introduction to Chaos. Pure and Applied Mathematics. Academic Press.

[50] Tomasetti C, Levy D (2010) Role of symmetric and asymmetric division of stem cells in developing drug resistance. Proceedings of the National Academy of Sciences 107: 16766–16771. 10.1073/pnas.1007726107 PMC2947914 20826440

[51] Berglund N, Gentz B (2006) Noise-induced phenomena in slow-fast dynamical systems: a samplepaths approach. Probability and its applications. Springer.

[52] Gardiner C (2009) Stochastic methods: a handbook for the natural and social sciences. Springer series in synergetics. Springer.

[53] Burness M, Sipkins D (2010) The stem cell niche in health and malignancy. In: Seminars in cancer biology. Elsevier, volume 20, 107–115. 10.1016/j.semcancer.2010.05.006 20510363

[54] Whiteside T (2008) The tumor microenvironment and its role in promoting tumor growth. Oncogene 27: 5904–5912. 18836471 10.1038/onc.2008.271 PMC3689267

[55] Maffini M, Soto A, Calabro J, Ucci A, Sonnenschein C (2004) The stroma as a crucial target in rat mammary gland carcinogenesis. Journal of cell science 117: 1495–1502. 14996910 10.1242/jcs.01000

[56] Gammaitoni L, Hänggi P, Jung P, Marchesoni F (1998) Stochastic resonance. Reviews of Modern Physics 70: 223.

[57] Van den Broeck C, Parrondo J, Toral R, Kawai R (1997) Nonequilibrium phase transitions induced by multiplicative noise. Physical Review E 55: 4084.

[58] Ridolfi L, D'Odorico P, Laio F (2011) Noise-Induced Phenomena in the Environmental Sciences. Cambridge University Press.

[59] Oksendal B (2003) Stochastic differential equations: an introduction with applications. Universitext (1979). Springer.

[60] Karlin S, Taylor H (2000) A second course in stochastic processes. Academic Press.

[61] Horsthemke W, Lefever R (1984) Noise-induced transitions: theory and applications in physics, chemistry, and biology. Springer series in synergetics. Springer.

[62] Kavinoky R, Thoo J (2008) The number of real roots of a cubic equation. The AMATYC Review 29: 3–8.

[63] Braumann CA (2007) Harvesting in a random environment: It or stratonovich calculus? Journal of Theoretical Biology 244: 424–432. 17070851 10.1016/j.jtbi.2006.08.029

[64] Kloeden P, Platen E (1992) Numerical solution of stochastic differential equations. Applications of mathematics. Springer-Verlag.

[65] Kalialis L, Drzewiecki K, Klyver H (2009) Spontaneous regression of metastases from melanoma: review of the literature. Melanoma research 19: 275. 19633580 10.1097/CMR.0b013e32832eabd5

[66] Da-jin W, Li C, Sheng-zhi K (1994) Bistable kinetic model driven by correlated noises: Steady-state analysis. Phys Rev E 50: 2496–2502. 10.1103/physreve.50.2496 9962285

[67] Atkinson R, Zhang M, Diagaradjane P, Peddibhotla S, Contreras A, et al (2010) Thermal enhancement with optically activated gold nanoshells sensitizes breast cancer stem cells to radiation therapy. Science translational medicine 2: 55ra79–55ra79. 10.1126/scitranslmed.3001447 PMC4123313 20980696

[68] Pietras A (2011) Cancer stem cells in tumor heterogeneity. Advances in Cancer Research 112: 256. 10.1016/B978-0-12-387688-1.00009-0 21925307

[69] Chow E (2012) Implication of cancer stem cells in cancer drug development and drug delivery. Journal of Laboratory Automation. 10.1177/2211068212454739 22893634

[70] Chen J, Li Y, Yu TS, McKay RM, Burns DK, et al (2012) A restricted cell population propagates glioblastoma growth after chemotherapy. Nature 488: 522–526. 22854781 10.1038/nature11287 PMC3427400

[71] Arens N, West I (2008) Press-pulse: a general theory of mass extinction? Paleobiology 34: 456–471.

[72] Driessens G, Beck B, Caauwe A, Simons BD, Blanpain C (2012) Defining the mode of tumour growth by clonal analysis. Nature 488: 527–530. 22854777 10.1038/nature11344 PMC5553110

