# Supplementary material for: A Possible Explanation for the Variable Frequencies of Cancer Stem Cells in Tumors
Source: PLoS One. 2013 Aug 7;8(8):e69131. doi: 10.1371/journal.pone.0069131 (PMC3737222; doi:10.1371/journal.pone.0069131)
Supplement: Appendix S1 — Appendix to a possible explanation for the variable frequencies of cancer stem cells in tumors. (PDF) [file pone.0069131.s001.pdf]

Appendix to
A possible explanation for the variable frequencies of cancer stem cells in tumors

Rescale transformations

In this appendix we detail the rescales made throughout the main text. The general model written in terms of the reactions is

$$
\begin{aligned}
C &\underset{k_2'/\Omega_2}{\overset{k_1}{\rightleftharpoons}} C + C \\
P &\underset{k_4'/\Omega_4}{\overset{k_3}{\rightleftharpoons}} P + P \\
C &\xrightarrow{k_5} C + P \\
C &\xrightarrow{k_6} P + P \\
P &\xrightarrow{k_7} \emptyset \\
P &\xrightarrow{k_8} C
\end{aligned} \tag{1}
$$

Using the law of mass action we have

$$
\begin{cases}
\dot{C} = k_1 C\left(1 - \frac{C}{\Omega_C}\right) - k_6 C + k_8 P \\
\dot{P} = k_3 P\left(1 - \frac{P}{\Omega_P}\right) + k_9 C - k_{10} P
\end{cases} \tag{2}
$$

with $k_9 \equiv k_5 + 2k_6$, $k_{10} \equiv k_7 + k_8$, $\Omega_C \equiv \frac{k_1}{k_2}$, $\Omega_P \equiv \frac{k_3}{k_4}$ and $k_2 \equiv k_2'/\Omega_2$, $k_4' \equiv k_4'/\Omega_4$. Using the reescale $C \equiv \Omega_C x$ and $P \equiv \Omega_P y$:

$$
\begin{cases}
\dot{x} = k_1 x\,(1 - x) - k_6 x + \frac{k_8 \Omega_P}{\Omega_C} y \\
\dot{y} = k_3 y\,(1 - y) + \frac{k_9 \Omega_C}{\Omega_P} x - k_{10} y
\end{cases} \tag{3}
$$

Using $t \equiv k_6 t'$ and $\Omega \equiv \frac{\Omega_P}{\Omega_C}$:

$$
\begin{cases}
\frac{dx}{dt'} = \frac{k_1}{k_6} x\,(1 - x) - x + \frac{k_8 \Omega}{k_6} y \\
\frac{dy}{dt'} = \frac{k_3}{k_6} y\,(1 - y) + \frac{k_9}{k_6 \Omega} x - \frac{k_{10}}{k_6} y
\end{cases} \tag{4}
$$

or

$$
\begin{cases}
x' = Ax(1 - x) - x + By \\
y' = Ey(1 - y) + Fx - Gy
\end{cases} \tag{5}
$$

with $x' \equiv \frac{dx}{dt'}$, $y' \equiv \frac{dy}{dt'}$ and

$$\begin{cases} A \equiv \frac{k_1}{k_6} \\ B \equiv \frac{k_2 k_3 k_8}{k_1 k_4 k_6} \\ E \equiv \frac{k_3}{k_6} \\ F \equiv \frac{k_1 k_4 k_9}{k_2 k_3 k_6} \\ G \equiv \frac{k_{10}}{k_6} \end{cases} \tag{6}$$

Gradient system

Starting from (30) and carrying out the transformation $C = s_1 c$, $P = s_2 p$ and $t = s_3 \tau$, we can write

$$\begin{cases} \frac{dc}{d\tau} = k_1 s_3 c \left(1 - \frac{s_1}{\Omega_C} c\right) - k_6 s_3 c + \frac{k_8 s_2 s_3}{s_1} p \\ \frac{dp}{d\tau} = k_3 s_3 p \left(1 - \frac{s_2}{\Omega_P} p\right) + \frac{k_9 s_1 s_3}{s_2} c - k_{10} s_3 p \end{cases} \tag{7}$$

Imposing $\frac{k_8 s_2 s_3}{s_1} = \frac{k_9 s_1 s_3}{s_2}$, $k_6 s_3 = 1$ and $s_1 = \Omega_C$, we obtain $s_1 \equiv \frac{k_1}{k_2}$, $s_2 \equiv \Omega_C \sqrt{\frac{k_9}{k_8}}$ and $s_3 = \frac{1}{k_6}$.

In this way we obtain

$$\begin{cases} \frac{dc}{d\tau} = \frac{k_1}{k_6} c (1 - c) - c + \frac{\sqrt{k_8 k_9}}{k_6} p \\ \frac{dp}{d\tau} = \frac{k_3}{k_6} p \left(1 - \frac{\Omega_C}{\Omega_P} \sqrt{\frac{k_9}{k_8}} p\right) + \frac{\sqrt{k_8 k_9}}{k_6} c - \frac{k_{10}}{k_6} p. \end{cases} \tag{8}$$

Potential $V(x,y)$

We want to write the equation (3) in the form $\dot{\mathbf{x}} = -\nabla V(x,y)$, where $\mathbf{x} = (x(t), y(t))^T$ (T means transpose), ∇ is the nabla operator. Integrating $f(x,y)$ from (3) with respect to x gives

$$V(x,y) = \int f(x,y) dx = -\frac{x^2}{2} + \frac{Ax^2}{2} - \frac{Ax^3}{3} + Bxy + f_0(y). \tag{9}$$

We must now obtain $f_0(y)$. Imposing $\partial_y V(x,y) = g(x,y)$, we obtain $Bx + f_0'(y) = g(x,y)$ and then $f_0(y) = \int [Ey(1-Fy) - Gy] dy = \frac{Ey^2}{2} - \frac{Gy^2}{2} - \frac{1}{3} EFy^3$. This provides the equation (5).
